# The Loneliness of the Odd One Out: How Deviations From Social Norms Can Help Explain Loneliness Across Cultures

**DOI:** 10.1177/17456916231192485

**Published:** 2023-10-11

**Authors:** Luzia Cassis Heu

**Affiliations:** Faculty of Behavioural and Social Sciences, Department of Interdisciplinary Social Science, Utrecht University

**Keywords:** loneliness, social norms, culture, norm deviations

## Abstract

Loneliness is an important health risk, which is why it is important to understand what can cause persistent or severe loneliness. Previous research has identified numerous personal or relational risk factors for loneliness. Cultural predictors, however, have been considered less. The new framework of norm deviations and loneliness (NoDeL) proposes that social norms, which are defining features of culture, can help explain loneliness within and across cultural contexts. Specifically, people who deviate from social norms are suggested to be at an increased risk for feeling lonely because they are more likely to experience alienation, inauthenticity, lower self-worth, social rejection, relationship dissatisfaction, and/or unfulfilled relational needs. Given that social norms vary by social, geographical, and temporal context, they can furthermore be considered cultural moderators between individual-level risk factors and loneliness: Personal or relational characteristics, such as shyness or being single, may increase the risk for loneliness particularly if they do not fit social norms in a specific environment. Integrating previous quantitative and qualitative findings, I hence offer a framework (NoDeL) to predict loneliness and cultural differences in risk factors for it. Thus, the NoDeL framework may help prepare culture-sensitive interventions against loneliness.

There were loads of people around me, but I felt misunderstood by all of them and hence lonely, because - I think that this is what loneliness is: If one feels misunderstood, or not accepted, or - wrong.

—Interviewee in in-depth interview about loneliness; Austrian man, age 35 (Heu, Hansen, et al., 2021)

Loneliness is an important risk factor for impaired mental and physical health (e.g., depression, anxiety disorders, cardiovascular disease, weakened immune system; S. Cacioppo et al., 2015). It is therefore relevant to understand what can cause and ease loneliness. In previous research, loneliness has typically been explained by individual or relational characteristics such as genetic disposition (e.g., Matthews et al., 2016), personality characteristics (Buecker et al., 2020), relationship status, or quality of social relationships (Hawkley et al., 2008). By contrast, deviations from social norms (i.e., unwritten rules about what is typically done and what ought to be done in a group; Cialdini et al., 1990; Eriksson et al., 2021) have been considered as risk factors for loneliness less (cf. e.g., Luhmann & Hawkley, 2016; Lykes & Kemmelmeier, 2014; Qualter et al., 2015).

This is surprising given that loneliness is commonly viewed as the unpleasant experience that flows from a perceived discrepancy between one’s actual relationships and relational ideals, needs, or expectations (Johnson & Mullins, 1987; Perlman & Peplau, 1981). Such relational ideals, needs, or expectations are influenced by what is socially normative (Akhter-Khan et al., 2023; de Jong Gierveld & Tesch-Römer, 2012; Johnson & Mullins, 1987; Luhmann & Hawkley, 2016; Lykes & Kemmelmeier, 2014; Perlman & Peplau, 1981). For instance, people in many cultures may feel lonely when alone on a Friday evening yet not on a Monday evening. They are neither likely to be more socially isolated on Friday than Monday evenings, nor would their individual desires change between Monday and Friday if they lived in a social vacuum. Instead, social norms prescribe to be with others on Friday yet not Monday evenings.

In this article, I propose the framework of norm deviations and loneliness (NoDeL), suggesting that loneliness can emerge if personal or relational characteristics do not fit in with a social environment. Specifically, the key idea in the NoDeL framework is that deviations from social norms increase the risk for feeling lonely because they can entail alienation, inauthenticity, lower self-worth, social rejection, relationship dissatisfaction, and/or unfulfilled relational needs. Although deviations from social norms are suggested to increase loneliness across cultures, the content of social norms varies by social (e.g., age, gender, socioeconomic status, political orientation), geographical (e.g., country, area, rural vs. urban area, different villages), and temporal context. As defining features of cultures (Chiu et al., 2010), social norms can thus help explain loneliness not only within but also across cultures: They can be considered cultural moderators between various individual-level risk factors and loneliness. Being single may, for example, more strongly relate to an increased risk for loneliness in cultures in which being in a partnership is more normative. Knowledge about social norms may then help better understand cultural variation in risk factors for loneliness, adding a macro- and exosystemic layer to common individual and microsystemic explanations for loneliness (in the bioecological model by Bronfenbrenner, 1979).

## Norm Deviations as Risk Factor for Loneliness

There are many different definitions of loneliness in the literature, which have in common that loneliness is viewed as a subjective (rather than objective) and unpleasant experience that revolves around perceived deficiencies in one’s social relationships (Bekhet et al., 2008; Heu, Hansen, et al., 2021). For instance, loneliness can be defined as result of a perceived discrepancy between actual and desired relationships (Perlman & Peplau, 1981), as feeling cutoff or separated from others (Hays & DiMatteo, 1987), or as perceived social isolation (VanderWeele et al., 2012). When laypeople are asked to define loneliness, they frequently describe feeling different from or misunderstood by others, such as in the following quote by a participant in qualitative research about meanings of loneliness in different cultures: “Well, really, [loneliness is] mostly a feeling of rejection, exclusion, not being understood, in a way losing the connection between you and a group of people, or all other people (Bulgarian man, age 33)” (Heu, Hansen, et al., 2021). Although there are numerous different reasons for feeling lonely (Heu, Hansen, et al., 2021), this definition already indicates that “not fitting in”—that is, deviating from social norms—can be an important aspect and predictor of the experience of loneliness.

Social norms trace what most people in a group are like or do (i.e., descriptive norms) or what they ought to be like or do (i.e., what most people in a group would approve or disapprove of; prescriptive or injunctive norms; Cialdini et al., 1990). Both prescriptive and descriptive social norms steer individual behavior and evaluations in a broad range of domains. They can, for example, influence whether shyness or assertiveness is more common (Chen, 2019) or how much alcohol people drink (Prentice & Miller, 1993). Nevertheless, some people will not (be able to) adhere to the social norms of their groups—a few out of protest but many rather involuntarily. For instance, people who do not find a suitable partner or prefer to remain single may not fit in with social norms to marry and have children; people who identify as nonbinary may not fit in with social norms to identify as man or woman. Such deviations from social norms have been discussed in relation to various outcomes, including impaired mental health and well-being (e.g., Higgins, 1989), social sanctions (e.g., Williams, 2002), or lower academic achievements (e.g., Oyserman et al., 2006), but rarely in relation to loneliness (cf. Lykes & Kemmelmeier, 2014).

However, not only previous loneliness conceptualizations (Perlman & Peplau, 1981) but also an evolutionary model of loneliness (J. T. Cacioppo et al., 2014) suggest that deviations from social norms can increase the likelihood of feeling lonely. From an evolutionary-psychological perspective, loneliness can be viewed as an evolved warning sign of (potential) social isolation. It is activated when individuals’ relationships risk to be deficient in quantity or quality and motivates to reconnect with others. After all, social isolation has been a threat to survival throughout most of human history. Not fitting in with social norms, then, seems likely to trigger loneliness because norm deviations are met with social sanctions across cultures (Eriksson et al., 2021), resulting in potential social exclusion. Indeed, brain regions that are typically active during error detection, conflict detection, or gap detection (e.g., between actual and desired states) seem to also be activated when individuals do not align with social norms or do not feel understood (Shamay-Tsoory et al., 2019). This may then signal the need to realign—for instance, with others’ behavior or emotions.

Although few studies have explicitly tested that deviations from social norms increase the likelihood to feel lonely, correlational studies have provided preliminary support. An age-normative life-stage perspective in developmental psychology proposes that the likelihood to feel lonely increases if individuals deviate from what is expected of someone their age (Luhmann & Hawkley, 2016; Qualter et al., 2015). Not having a romantic relationship, for example, was found to be more strongly related to higher loneliness among middle-aged adults than among young or older adults in a German sample (Luhmann & Hawkley, 2016)—arguably because being in a partnership is more common in midadulthood than in young or late adulthood. Furthermore, in many cultures, building up own social networks is a normative developmental task in adolescence and young adulthood (Qualter et al., 2015). Accordingly, having more social relationships and more frequent social contact were more strongly related to less loneliness among younger than older adults in American and British samples (Green et al., 2001; Victor & Yang, 2012).

Further empirical support comes from studies that compare associations between individual-level characteristics and loneliness in different geographical contexts. Whereas people in more individualistic cultures tend to establish own social networks outside the family when growing up, kin relationships often remain central in more collectivistic cultures (Triandis, 1995). Being embedded in mutually caring relationships hence seems more socially normative, implying that individuals who lack family relationships in collectivistic cultures may perceive larger discrepancies between their actual and desired social relationships than in individualistic cultures (Lykes & Kemmelmeier, 2014). Indeed, lacking interaction with family was found to be more strongly related to loneliness in more collectivistic European countries (e.g., Bulgaria, Portugal) than in more individualistic European countries (e.g., the Netherlands, the UK; Lykes & Kemmelmeier, 2014). Likewise, deviations from normative family transitions (e.g., never having lived with a partner, remaining childless, or having children later than most others) were more strongly related to loneliness among older adults in European countries in which traditional family relations were more normative (Zoutewelle-Terovan & Liefbroer, 2018).

By contrast, romantic relationships seem to sometimes be construed as more desirable and more relevant sources of psychological intimacy in more individualistic cultures such as in the United States than in more collectivistic cultures such as in South Korea (Seepersad et al., 2008). Accordingly, in comparison with young adults in romantic relationships, American young adults without a romantic relationship reported more romantic loneliness than South Korean young adults without a romantic relationship.

Finally, it seems that more instrumental support (i.e., provision of practical help, including help with chores and errands) is exchanged in interdependent cultures of southern Europe, whereas more emotional support (i.e., provision of sympathy, appreciation, or approval) is exchanged in independent cultures of northern Europe (Sánchez Rodrigues et al., 2014). In line with the idea that deviations from social norms increase the risk for loneliness, providing and receiving instrumental support was more strongly associated with less loneliness in a Spanish sample, whereas receiving emotional support was more strongly associated with less loneliness in a Dutch sample.

Studies examining change of social norms over time also indicate that norm deviations may imply more loneliness: Throughout the second half of the 20th century, living alone became more common among elderly people in the UK and, at the same time, less strongly related to more loneliness (Victor et al., 2002). Furthermore, in Western cultures, shyness tends to be viewed rather negatively and has been found to relate to impaired psychological adjustment (Chen, 2019), including stronger loneliness (Jackson et al., 2002; Tian et al., 2019). However, in traditional Chinese culture, shyness is accepted and even appreciated and was thus associated with positive psychological adjustment in different Chinese studies conducted in the 1990s (for an overview, see Chen, 2019). Relatedly, more shyness predicted stronger loneliness among Brazilian and Italian children yet not Chinese children (Chen et al., 2004). Only with a cultural shift toward a more competitive, market-oriented culture as typical in Western countries, shyness was, in more recent studies, related to lower well-being in urban (yet not in rural) China (Chen, 2019).

In sum, these studies suggest that nonnormative characteristics may increase the risk for loneliness. In addition, they highlight that associations between individual-level characteristics and loneliness differ between contexts with supposedly different social norms. This indicates that social norms may moderate associations between different individual-level characteristics and loneliness.

## The NoDeL Framework

**Fig. 1.**
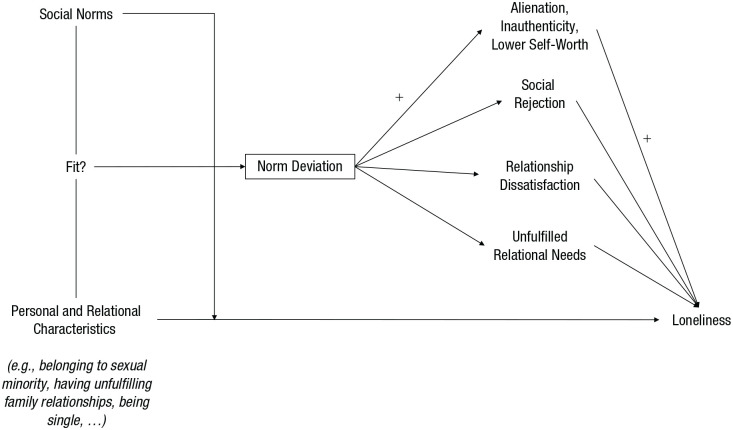
The framework of norm deviations and loneliness.

**Fig. 2.**
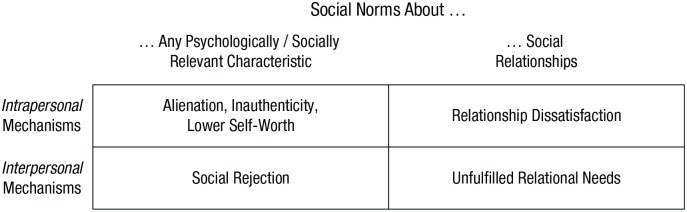
Mechanisms in the norm deviations and loneliness framework.

Expanding on previous correlational research, the NoDeL framework proposes that norm deviations can cause stronger loneliness through different intrapersonal and interpersonal mechanisms: alienation, inauthenticity, lower self-worth, social rejection, relationship dissatisfaction, and/or unfulfilled relational needs (see right side of Fig. 1; Fig. 2). These mechanisms are independent from each other. They do not exclude each other, can occur simultaneously, and may often entail each other. For instance, social rejection can imply blocked access to relational provisions. Feeling alienated from others may result in unfulfilled relational needs because individuals who experience alienation are more likely to socially withdraw (Watson & Nesdale, 2012).^1^ Given that the mechanisms in the NoDeL framework depend on different preconditions (see Table 1), a broad range of deviations from social norms can increase the risk for loneliness. This section, therefore, serves to clarify assumptions and scope of the NoDeL framework before I describe each mechanism in more detail in the next section.

**Table 1.** Prerequisites for Different Mechanisms in the Norm Deviations and Loneliness Framework

	Alienation	Inauthenticity	Lower self-worth	Social rejection	Relationship dissatisfaction	Unfulfilled relational needs
Norm deviation needs to reflect objective reality						x
Norm deviation needs to be						
. . . perceived by individual who deviates	x	x	x		x	
. . . perceived by others				x		
Norm deviation needs to be						
. . . relevant to individual who deviates (psychologically relevant)	x	x	x		x	
. . . relevant to others (socially relevant)				x		
Social norm needs to be internalized						
. . . by person who deviates			x		x	
. . . by others						
Long-lasting deviation is required						x
Deviation from prescriptive norm is required			x		x	x

Alienation, inauthenticity, lower self-worth, and relationship dissatisfaction emerge in people’s minds, whereas social rejection and unfulfilled relational needs emerge in their social relationships (see rows of Fig. 2). The comparison between an individual’s characteristics with social norms, which is illustrated by the left side of Figure 1, can thus occur in the mind of individuals who perceive to deviate themselves, in the mind(s) of others around them, or reflect a more objective reality. Relatedly, as illustrated in Table 1, only unfulfilled relational needs require that individuals actually deviate from social norms. All other mechanisms in the NoDeL model rely on subjective perceptions of norm deviations rather than objective realities. Indeed, individuals seem to be influenced by and act in line with what they believe others are like even if such beliefs are incorrect (i.e., pluralistic ignorance; Allport, 1924; Prentice & Miller, 1993).

Relationship dissatisfaction and unfulfilled relational needs can be triggered only by deviations from norms about social relationships (see columns in Fig. 2), but other mechanisms can also be triggered by deviations from norms about different personal characteristics, such as emotions, interests, or preferences (cf. de Jong Gierveld & Tesch-Römer, 2012). This goes beyond the widespread conceptualization of loneliness as result of a discrepancy between individuals’ actual and desired social relationships (Perlman & Peplau, 1981). However, people who are unemployed, are homeless, are physically disabled, have a psychiatric diagnosis, are part of a sexual minority, or have stigmatized illnesses are typically found to have relatively high levels of loneliness (Lindgren et al., 2014; Pedersen et al., 2012; Rokach, 2014). In addition, Belgian students who experienced more negative emotions, such as sadness or anxiety, reported more loneliness if they perceived that society disapproved of rather than accepted such emotions (Bastian et al., 2015). Likewise, in samples from the United States, Belgium, and Korea, experiencing different emotions than others in one’s cultural context was found to be related to lower self-reported relational well-being (De Leersnyder et al., 2014), which can, in turn, be regarded as risk factor for loneliness (Hawkley et al., 2008; Perlman & Peplau, 1981).

This is not to say that any norm deviation can trigger mechanisms in the NoDeL framework.^2^ Most deviations from social norms need to be either psychologically or socially relevant (or both) to increase the risk for loneliness. Social relevance describes that adherence to a norm is considered relevant by different members of a group and is, thus, important for group inclusion or status within the group. Normative characteristic may, for example, become socially relevant if they are central to social identity (Tajfel & Turner, 1982). Whereas social relevance should be key to interpersonal mechanisms such as social rejection (Bennett, 2014), intrapersonal mechanisms, rather, rely on psychological relevance. Psychological relevance describes that an individual—in the NoDeL framework, the person who deviates—perceives a norm deviation to be socially relevant. Psychological relevance is likely to emerge only in groups in which inclusion and opinions of others matter to an individual (i.e., reference groups; Sherif & Sherif, 1964). Ultraorthodox Jewish men, for example, seemed to experience ostracism by ultraorthodox Jewish women as less distressing than ostracism by other ultraorthodox Jewish men (Yaakobi, 2021). Indeed, gender segregation in ultraorthodox Jewish communities may culturally justify not being part of a group of the opposite gender, eliciting weaker psychological reactions than exclusion by members of the same gender.

Whereas most mechanisms require that social norms are relevant to some actor in the NoDeL model, only mechanisms of self-worth and relationship dissatisfaction rely on the internalization of social norms. Internalization describes that individuals privately adopt opinions, attitudes, values, or behavior that they perceive as normative among others around them. Self-worth has, for example, been suggested to be influenced by comparisons between actual selves and internalized standards (self-discrepancy theory; Higgins, 1989). Relatedly, effects of upward social comparison on body dissatisfaction appeared to be stronger among women who internalized thin ideals (Myers et al., 2012). By contrast, alienation, inauthenticity, or social exclusion are independent from internalization: People may feel alienated from others who drink on a night out even if they do not privately believe that one should drink. People may exclude an abstinent individual because they believe that other group members expect drinking even if they do not appreciate it themselves. Although psychological relevance and internalization may seem strongly related to each other, they are conceptually distinct: For instance, an individual may perceive that drinking alcohol is relevant for belonging to a group of friends without privately agreeing that it is desirable to drink. Such psychological relevance without internalization may then result in alienation and inauthenticity yet less likely in lower self-worth.

Deviations from social norms can be brief (e.g., being the only married person at a party) or longer lasting (e.g., having a different political orientation than others in one’s study program). Although longer-lasting or repeated experiences of deviating from norms may more often result in chronic loneliness (i.e., long-lasting loneliness; de Jong Gierveld & Raadschelders, 1982) than brief deviations, even just brief norm deviations should trigger the mechanisms in the NoDeL framework. Disagreeing with others’ political opinion at a dinner party, for example, may make individuals feel disconnected or like they cannot express their authentic opinion (i.e., alienation, inauthenticity). They may conclude that their relationships are not as good as they would want them to be (i.e., relationship dissatisfaction), or they may be ignored by or gossiped about by others if they express their deviating opinion (i.e., social rejection). Nevertheless, personal sensitivity to deviating from social norms may turn even just brief deviations into chronic loneliness (Watson & Nesdale, 2012; see “Practical Implications”).

Because the main premise of the NoDeL framework is that being or feeling different from others can cause loneliness, descriptive norms seem more central in the NoDeL framework than prescriptive norms. Indeed, people who deviate from descriptive norms can experience alienation or inauthenticity even if they do not deviate from prescriptive norms. For instance, people who experience less marital problems, have more friends, or are more outgoing than others may not share others’ experiences and, therefore, feel more alienated. In addition, they may conceal their achievements or desirable characteristics to avoid “feeling too different to get along” (Arnett & Sidanius, 2018, p.109), resulting in lower authenticity. However, relationship dissatisfaction, lower self-worth, and social rejection require that individuals deviate from social norms that are both descriptive and prescriptive. For these mechanisms, individuals do not only need to be different from others, but even more importantly, they also need to be perceived as wrong (i.e., a deviation from a prescriptive norm). In addition, in practice, boundaries between descriptive and prescriptive norms are often blurred because laypeople tend to assume that what most people do is also what should be done or that what should be done is also what most people do (Eriksson et al., 2015).

## Mechanisms Linking Norm Deviations and Loneliness

In this section, I describe the mechanisms in the NoDeL framework in more detail and illustrate them with quotes from qualitative research (Heu, Hansen, et al., 2021): In 42 semistructured in-depth interviews from India, Egypt, Israel, Bulgaria, and Austria, people between the age of 24 and 45 years provided loneliness definitions, causes, and remedies. Participants were asked to talk about their most recent loneliness experience, and norm deviations emerged as a recurrent topic (for a more detailed description of study design, method, and results, see Heu, Hansen, et al., 2021).

### Alienation, inauthenticity, and lower self-worth

For one, when people perceive to deviate from social norms themselves, they may be more likely to feel lonely because of feeling alienated or disconnected from others. Members of marginalized groups, for example, have been suggested to often “feel different, misunderstood, or estranged from others” (Elmer et al., 2022, p. 2271), and these experiences have been described as causes of loneliness in the qualitative interviews from Heu, Hansen, et al. (2021):

Well, I think it’s - it’s the worst if one feels misunderstood. And when one is, for instance, in a group - that can be with pupils or colleagues, that can be family. . . . And you are the only one who is of an opinion or conviction, and everyone else is not. . . . Then that can indeed trigger the feeling of loneliness. (Austrian woman, age 35)

Alienation is closely related to not feeling understood, which can emerge if others do not express appreciation or validation of what a person experiences or is like (Reis & Shaver, 1988). Not feeling understood is then pivotal for loneliness (Heu et al., 2019; Perlman & Peplau, 1981)—among others because it has been found to be key to relationship satisfaction and feeling connected (e.g., Oishi et al., 2010; Reis & Patrick, 1996). Indeed, participants in qualitative interviews from Heu, Hansen, et al. (2021) reported feeling lonely when they deviated from social norms because others appeared not to understand their perspective:

Yes, you see, and there my ADHD comes in, right? Erm, I’ve always had the feeling that I was a little different, right? And there it was already lonely because people didn’t understand what I explained [right?], what I want to tell them. They didn’t understand [right?] that I think differently, that I am different [right?]. That I feel differently. (Austrian man, age 41)

I don’t like to drink, I don’t like to party. . . . For example, there’s gonna be a promotion party, . . . then I’ll usually end up sitting in a corner, and - having uh - having Sprite or Coke or something - anything - pizza, whatever - by myself. So, I feel very lonely at that point. (Indian man, age 28)

I remember that, during my time at the military base, I felt really lonely because I was a “dos” [religious Jew] within a military base full of secular guys, and some gentiles [non-Jews]. . . . It was a very difficult service in this respect, up until I found some normal guys to hang out with. (Israeli man, age 37)

Second, if individuals’ private attitudes or tendencies do not fit social norms, they may not be able to be authentic around others (Schmader & Sedikides, 2018). However, being authentic has been suggested to be a basic relational need (Zvelc et al., 2020), and unfulfilled relational needs can make people feel lonely. Indeed, in qualitative interviews, deprived youths in the UK repeatedly mentioned lack of authenticity in relationships as cause for loneliness (Tagomori et al., 2022). Little opportunity to be authentic is also central in the quote below, in which the participant perceived a mismatch between her own and others’ emotional experiences:

I don’t want to compare my problems with other people, but they don’t seem to be that sad [laughs] half the time. If they are, then they don’t show it, and I don’t want to be the only sad person in the room. So . . . it takes a lot of toll on you if you pretend all the time. And therefore, I can’t be around people, I keep pretending. (Indian woman, age 24)

Relatedly, a phenomenological perspective on loneliness suggests that people feel lonely if their self-presentations do not converge with their private self-perceptions (Whitehorn, 1961) or if they hide their true self behind a social façade (Rogers, 1970). For instance, stigmatized groups are often reluctant to disclose information about their personal lives, implying more distant interactions and less connectedness (Newheiser & Barreto, 2014). Concealing sexual orientation as a member of a sexual minority, for example, was found to relate to stronger loneliness (Elmer et al., 2022; Huang & Chan, 2022; Jiang et al., 2019).

Third, deviations from social norms may increase loneliness through lower self-worth. Indeed, individuals who deviate from social norms may feel less valuable than individuals who conform because they compare their own with others’ characteristics (social-comparison theory, Festinger, 1954; self-discrepancy theory, Higgins, 1989). In an experimental study among American university students, for example, more comparisons on social media with others who were perceived to be doing better predicted lower self-esteem (Vogel et al., 2014). Even just having different personality characteristics or a different level of religiosity than others in one’s city was related to lower self-esteem in an American study about person-environment fit (Bleidorn et al., 2016). Accordingly, members of stigmatized groups seem to internalize negative societal attitudes about themselves (Elmer et al., 2022).

Perhaps unintuitively, lower self-worth can then increase the risk of feeling lonely (Heu, Hansen, et al., 2021; Qualter et al., 2013; Rokach, 1989). In an English study, for example, lower self-worth at age 5 predicted higher levels of loneliness between ages 7 and 17 (Qualter et al., 2013). Furthermore, in a British sample, more comparisons with others on Facebook who were perceived to be doing better were related to stronger loneliness (Dibb & Foster, 2021). A participant in the Heu, Hansen, et al. (2021) qualitative research described the link between a troubled relationship with oneself and loneliness as follows:

The loneliness came from the inside, the outside is not . . . like, it’s something that I have with myself – the difficulty of being alone with myself in peace. So, the environment is distracting, but it’s not solving the problem. . . . When you are in a relationship, it’s – you let somebody else accept you, love you, you know – you don’t have to do it yourself. But, you know, that’s something I learned through the years, that, if you don’t do it yourself, it won’t come from outside. (Israeli woman, age 33)

Deviations from social norms may hence increase the risk for loneliness through low-quality relationships not only with others but also with oneself (Heu, Hansen, et al., 2021).

### Social rejection

When you feel rejected, rejected from the community, rejected from other people. That is when you feel lonely. You can feel misunderstood or excluded from the community in many ways. And that is when loneliness occurs. (Bulgarian man, age 33)

Perhaps most obviously, people who deviate from social norms are more likely to feel lonely because of a higher risk to be ostracized (i.e., excluded, ignored, or rejected; Williams, 2002), gossiped about (i.e., others talk about their norm deviation), or confronted about their norm deviation (Eriksson et al., 2021; Gelfand et al., 2006; Hitti et al., 2011; Korkiamäki, 2014):

I’m a teacher and sometimes I have slightly non-standard ideas about how the classes should go - about the communication with students. Some people react strangely and when I am with more than two or three people like that, I don’t feel quite well. Like, alone, isolated, misunderstood. (Bulgarian woman, age 44)

[I felt lonely] when I had a few hiccups in my business and people who were close to me started distancing themselves from me and didn’t want to associate with me. At that time, friends in my circle and relatives distanced themselves [from me]. (Indian man, age 45)

When you’re good, like, when you’re doing well, no?, like everybody comes to you. But when you’re not doing well, no?, it’s like, turns opposite. Then you cannot rely on anybody. (Indian woman, age 38)

If I am doing anything wrong, nobody will be with me. Drinking, roaming and fighting around here and there - nobody will let me be around them. They’ll say, “hey, this guy is strange.” They will scold me and send me away. . . . If they think that you are bad, nobody will want to come and talk. (Indian man, age 27)

More specifically, interpersonal consequences of norm deviations range from open rejection and exclusion from a community (e.g., Eriksson et al., 2021) to subtle withdrawal such as emotionally distancing oneself or preventing moving from small talk to authentic conversations. For instance, members of sexual minorities have been described to be confronted not only with overt hostility (e.g., discrimination, verbal harassment, and violence) but also with more subtle or ambiguous communication of disapproval, disrespect, or hostility, implying more loneliness (Elmer et al., 2022):

We have a WhatsApp group of the whole class, and then there is a group for the guys and the girls. And I’m in neither of them. Now, it’s funny because we are in 2019. So, like, the boundaries should’ve blurred a long time ago. But the group of the boys - I’m not in there. Not because, like, they defined me intentionally, but they know that I won’t go to a lake with them and I won’t play football with them. . . . And the girls also do, like, girls’ nights. And I understood that there was a discussion about: “Should we add [participant name] to sushi nights?” and all the other things that they do. They didn’t add me and it’s fine like that. . . . And I just think that I felt really alone in the sense that I felt like I am the different one every time people saw me as that different one and treated me like it. (Israeli man, age 26)

Accordingly, social rejection is likely to (partly) explain relatively high levels of loneliness among members of groups that are stigmatized, marginalized, and/or experience discrimination, such as ethnic minorities (Juang & Alvarez, 2010; Lee & Turney, 2012; Priest & Williams, 2018), sexual minorities (e.g., Elmer et al., 2022), people suffering from mental illness (Świtaj et al., 2015), or migrants (Wang et al., 2021).

I felt very lonely there [at school] because I was a foreigner . . . and others made me feel that. I don’t believe that the [other] children did this on purpose, but society does - and children hence do that, too. (Austrian man, age 35)

Note that loneliness itself is often stigmatized (Barreto et al., 2022), implying that being lonely may, ironically, also increase the risk for loneliness.

Although in this section I mostly discussed the consequences of actual social rejection, the mere perception of being rejected (i.e., “not ostracism” in Williams, 2002) can also increase the risk for loneliness (Vanhalst et al., 2013). People who deviate from social norms may more often interpret others’ behavior as rejection (Feinstein, 2020) because both previous experiences of rejection and loneliness make people more likely to detect rejection and/or erroneously perceive to be rejected (i.e., rejection sensitivity; Downey & Feldman, 1996; Spithoven et al., 2017). Rejection sensitivity was, in turn, found to be associated with more social withdrawal (Watson & Nesdale, 2012), rejecting-defensive behavior that can deter others (for an overview, see Elmer et al., 2022), lower relationship quality (Norona et al., 2018), and alienation (London et al., 2012). Thus, perceiving to be rejected by others seems to imply a higher risk of feeling lonely (Gao et al., 2017) irrespective of whether others actually reject or not.

### Relationship dissatisfaction

Loneliness has often been defined as the unpleasant experience that emerges if individuals’ social relationships are different from their desired relationships (Perlman & Peplau, 1981). Because desired relationships are influenced by social norms, relationships that do not live up to social norms can cause relationship dissatisfaction, increasing the risk for loneliness (Akhter-Khan et al., 2023; de Jong Gierveld & Tesch-Römer, 2012; Heu et al., 2019; Johnson & Mullins, 1987; Luhmann & Hawkley, 2016; Lykes & Kemmelmeier, 2014; Perlman & Peplau, 1981). For instance, American and English adults who compared themselves with others with better social contact reported more loneliness than adults who compared themselves with others with worse social contact (Arnold et al., 2021). Multiple participants in the Heu, Hansen, et al. (2021) qualitative interviews reported having felt lonely when single—not necessarily (only) because they missed having a partner but because they perceived to deviate from the norm to be in a romantic relationship:

There have been moments in which not being in a relationship has been pressuring me and has made me feel lonely. (Bulgarian man, age 33)

Well, the comparison with everyone else. It’s like, why does everyone else manage [to establish a romantic relationship], why don’t I, you see? (Austrian man, age 33)

Because you’d be going outside, you’d be seeing different sorts of people and uh- sometimes, . . . you would have wished that you had uh you had a boyfriend or a girlfriend. (Indian man, age 28)

### Unfulfilled relational needs

Different from relationship dissatisfaction, which emerges in people’s minds, unfulfilled relational needs result from actual shortcomings in people’s relationships. Human beings have been suggested to have different relational needs, such as for instrumental assistance, advice, reassurance of worth, emotional closeness, belonging, or being needed by others (Weiss, 1974). A different taxonomy by Erskine et al. (1999) includes needs for security; to feel validated, affirmed, and significant; to be accepted by a stable, dependable, and protective other; to self-define in relation to others; to have personal experience confirmed; to have impact on another person; and to express love.

Social norms define in which relationships such relational needs are catered to. For instance, partnerships are, in many societies, main sources of emotional and physical closeness (e.g., Burleson et al., 2019; Seepersad et al., 2008). Lacking a partnership as a socially normative relationship can then reduce or block access to relational provisions, resulting in more loneliness (Weiss, 1973). People who are divorced, widowed, or single may, among others, be deprived of physical touch or opportunities to discuss private thoughts and emotions (e.g., Jakubiak & Feeney, 2017)—particularly if other social norms prevent need fulfillment in different relationships. Traditional gender norms, for example, often do not allow as much emotional or physical closeness in male friendships as in female friendships (Lewis, 1978; Scoats & Robinson, 2020). Single men may hence be more likely not to have their relational needs fulfilled than single women or men in cultures in which emotional and physical closeness are also normative in male friendships:

So, I was looking for something, for example a marriage relationship, and in such an oriental and Islamic society, you seek marriage because it’s the only way to live freely with a partner whom I can open up to and be my real self with. Because when you show your real self to someone, it feels good - especially if they are trustworthy of your secrets. And this is why I was looking for a relationship (partnership): because there was no close friend who I could trust enough to talk to him about all my feelings. (Egyptian man, age 33)

Likewise, family relationships are often central sources of instrumental support. Individuals who come from a conflicted family background, who have not started an own family, or who have lost family members can, among others, lack help when moving houses or when needing someone to do groceries when they are ill. The quote below was shared by a participant in a small Bulgarian town (i.e., arguably a more family-oriented culture) who had recently lost multiple family members:

Let’s say on holidays, in moments where you’re used to gathering with your family, there comes a point when you no longer have someone to gather with. . . . I mean, friends invite you over, you spend those moments with them, but it is not your family. (Bulgarian woman, age 44)

Finally, in Western middle-class families, it is mostly one or two parents who take care of children, whereas in most less industrialized cultures, siblings and extended kin (e.g., grandparents, aunts, uncles) are also strongly involved in child rearing (Sear, 2016). Thus, childless adults in cultures in which parents are main caregivers may more often lack opportunities to take care of others than in cultures in which communal caregiving or alloparenting are common. This is relevant because taxonomies of relational needs include caring for others (Erskine et al., 1999; Weiss, 1974), and not having a person to care for may therefore entail loneliness (Weiss, 1973).

Note that the absence of a certain type of relationship may increase loneliness through different unfulfilled needs in different cultures. In West African cultures, for example, instrumental support (e.g., help with moving houses) seems to typically be offered by friends (Adams & Plaut, 2003). By contrast, in European or North American cultures, instrumental support is, rather, provided by family members or romantic partners, whereas friends are mostly expected to offer emotional support. Although not having friends may hence imply lacking someone to help repair a car in a West African culture, it may imply lacking someone to talk to about one’s worries in a Western European culture. Nevertheless, both unfulfilled relational needs may increase the risk for loneliness (Sánchez Rodrigues et al., 2014; Weiss, 1974; Wolfers et al., 2022). In sum, not having a specific type of relationship (e.g., a partnership) may not universally imply a lack of relational provisions and loneliness. However, not having relationships that social norms define to be crucial sources of relational provisions is likely to foster loneliness.

## The NoDeL Framework Across Cultures

People across different cultures feel lonely (van Staden & Coetzee, 2010), compare themselves with others (Baldwin & Mussweiler, 2018), conform to social norms (Bond, 2004; Bond & Smith, 1996), socially sanction people who deviate from these norms (Eriksson et al., 2021), and suffer when they are socially rejected (Uskul & Over, 2017; Yaakobi, 2021). Accordingly, it seems plausible that deviating from social norms increases loneliness across cultures and that most of the broad mechanisms in the NoDeL framework are cross-culturally similar. Indeed, in qualitative interviews (Heu, Hansen, et al., 2021), individuals in quite different cultural contexts, such as India and Austria, reported feeling lonely when their relationships did not live up to their own or others’ expectations (relationship dissatisfaction in the NoDeL framework); because of feeling different from, misunderstood by, or unable to be authentic around others (alienation, inauthenticity); when lacking a good relationship with themselves (lack of self-worth); when some of their relational needs, such as for being supported or for closeness, were not fulfilled (unfulfilled relational needs); or because of being excluded (social rejection; Heu, Hansen, et al., 2021).

Exactly because of these consistencies, the NoDeL framework may help analyze cultural differences in predictors of loneliness across cultures. If deviations from social norms increase loneliness across different cultures while social norms—as defining aspect of culture—vary between social contexts, the individual characteristics that increase the risk for loneliness should differ (for examples of cultural variation in relationship expectations, see e.g., Akhter-Khan et al., 2023). As illustrated on the left side of Figure 1 and suggested by research on correlates of loneliness across different social contexts (e.g., little interaction with family, Lykes & Kemmelmeier, 2014; living alone, Victor et al., 2002; being single, Seepersad et al., 2008), social norms can hence act as cultural moderators in the association between different individual-level characteristics and loneliness (as suggested for relationship characteristics by de Jong Gierveld & Tesch-Römer, 2012). This is not to say that risk factors for loneliness are likely to differ fundamentally: In qualitative interviews, broad reasons for feeling lonely seemed similar across samples from different cultural contexts (Heu, Hansen, et al., 2021). Nevertheless, social norms seem to potentially alter how strongly certain risk factors can predict loneliness.

### Variability of the NoDeL framework across cultures

According to the NoDeL framework, norm deviations increase the likelihood of feeling lonely across cultures. Nevertheless, the share of variance in loneliness that the NoDeL framework can explain may differ across cultures and social contexts. For instance, norm deviations may more strongly predict loneliness if adhering to social norms is culturally more important (i.e., in tighter cultures; Gelfand et al., 2006). Likewise, norm deviations may more strongly influence loneliness among adolescents and young adults than among older age groups because youths seem to be more sensitive to social norms and rejection (Goossens, 2018; Norona et al., 2018; Steinberg & Monahan, 2007). Brain regions involved in emotion processing of rejection and inclusion and those involved in thinking about others’ reactions and thoughts seem more active among adolescents than in other age groups (for a review, see Goossens, 2018).

Moreover, the relative frequency with which each mechanism in the NoDeL framework occurs and their psychological impact (i.e., how strongly they relate to loneliness) may differ. Social rejection, for example, may more commonly link norm deviations to loneliness in more collectivistic cultures, such as in Ghana, Indonesia, or Colombia. In such cultures, ostracism or confrontation for norm deviations have been found to be more common than in more individualistic cultures, such as in Australia, the UK, or the Netherlands (Eriksson et al., 2021). Furthermore, social rejection may more strongly predict loneliness among adolescents than in older age groups as adolescents seem to be relatively susceptible to social rejection (e.g., Goossens, 2018).

Finally, although the mechanisms identified in the NoDeL framework are likely to occur in a broad range of cultures, they may manifest in culture-specific concrete situations and behavior. For instance, what exactly social rejection looks like should vary with differences in how social norms are typically enforced (Eriksson et al., 2021): Members of some cultures may tend to openly tell others that their behavior is wrong (e.g., “You need to eat less, you have gained weight.”), whereas members of different cultures may, rather, ask questions (e.g., “Don’t you get bored when you travel all by yourself?”). Likewise, different types of groups can serve as reference groups (i.e., groups in which norm deviation can become psychologically relevant and hence trigger mechanisms in the NoDeL framework). Exclusion by strangers was, for example, found to have a stronger psychological impact on members of herding cultures than on members of farming cultures, arguably because members of herding cultures depend more strongly on help by strangers (Uskul & Over, 2017). A test of the NoDeL framework, may, consequently, require different operationalizations of concepts and mechanisms in which questions, items, or scenarios, for example, reflect the cultural realities people live in.

## Discussion

Most research and theorizing about loneliness have focused on relational or personal loneliness risks such as few or low-quality relationships, few social interactions, being unmarried, neuroticism, or genetic disposition (for an overview, see Heu, Hansen, et al., 2021). The NoDeL framework adds a macro- and exosystemic layer to such individual and microsystemic explanations (in the bioecological model by Bronfenbrenner, 1979). It proposes that deviating from social norms increases the risk for feeling lonely. Thus, social norms, which vary by social, geographical, and temporal context (Chiu et al., 2010), can act as cultural moderators in associations between individual-level characteristics and loneliness. The NoDeL framework hence allows to predict loneliness within different cultures and to analyze the risk potential of individual-level characteristics across cultures.

### Theoretical implications

Brief loneliness when deviating from social norms may be viewed as evolutionarily developed warning sign of potential social exclusion. Long-lasting or repeated norm deviation may, however, result in chronic loneliness. Consequently, the NoDeL may help identify groups that are at a higher risk for chronic loneliness. For instance, people who belong to a stigmatized or marginalized group may be particularly at risk for chronic loneliness because they will repeatedly experience both intrapersonal and interpersonal mechanisms described in the NoDeL framework. This aligns with predictions by minority-stress theory, which explains poorer mental-health outcomes among stigmatized groups by external processes, such as discrimination, harassment, and violence, and internal processes, such as concealment, self-stigma, or rejection sensitivity (Meyer, 2003).^3^ Recently, minority-stress theory was proposed to also explain stronger loneliness among sexual-minority adults from 85 countries (Elmer et al., 2022).

In addition, the risk for chronic loneliness may be increased for people who are comparatively sensitive to social rejection—for instance, because they were socially rejected earlier in their lives or feel lonely (J. T. Cacioppo et al., 2014; Watson & Nesdale, 2012). Accordingly, they may more easily perceive to deviate from social norms and thus experience the resulting loneliness more frequently. Some people may also be genetically predisposed to feel different from or rejected by others. After all, between 30% and 40% of the variation in related phenomena such as loneliness or depression have been found to be explainable by genetic disposition (Matthews et al., 2016).

Finally, deviating from social norms can cause chronic loneliness if personal or contextual restrictions prevent individuals from leaving situations in which they do not fit in. Certain people may, for example, be more hesitant to create new social relationships because of social anxiety. They may also be more reluctant to leave well-known social contexts for fear of becoming socially isolated. Furthermore, people cannot simply seek new groups in cultures in which it is uncommon to create new social relationships (Yuki & Schug, 2012) or in cultures in which the range of acceptable behavior or characteristics is very narrow (Heu, van Zomeren, & Hansen, 2021). In cultures in which most people get married because this is the only socially acceptable life path, for example, people who do not find a suitable partner cannot simply move to a social context in which they fit in better. They may thus more often be pushed into chronic loneliness.

Additional to helping explain some forms of chronic loneliness, the NoDeL framework also suggests that fit between personal and environmental characteristics should be more strongly considered when predicting loneliness (e.g., in polynomial regressions or response surface modeling; Rauthmann, 2021). Currently, individual- and culture-level risk factors are mostly examined separately, and risk factors are often assumed to be universal. This seems problematic because most psychological research, including research about loneliness, has been conducted among undergraduate students in the Global North (Henrich et al., 2010). Risk factors that have been identified in these studies may, therefore, be less relevant in other social or geographical contexts.

When exploring relevant loneliness risks in a cultural context, a first step could thus be to collect information about social norms—for instance, from existing scientific literature (e.g., Adams & Plaut, 2003; Argyle et al., 1986), qualitative research, or cultural products (e.g., schoolbooks, advertisement, [social] media; Morling & Lamoreaux, 2008). An analysis of Hollywood movies, for example, may reveal that emotional or physical closeness were, at least until recently, not normative in male friendships in the United States (Scoats & Robinson, 2020). This suggests that men who were single or preferred closeness in their friendships may have been more likely to feel lonely—for example, because they were not able to fulfill relational needs, were rejected by friends when seeking closeness, or were socially sanctioned for displaying closeness in friendships (e.g., parental disapproval, gossip). Knowledge about social norms and about what constitutes a norm deviation may thus help identify risk factors for loneliness and, accordingly, identify groups that may require a loneliness intervention.

### Practical implications

One practical takeaway from the NoDeL framework is that the relevance of loneliness causes can vary cross-culturally and that interventions may hence address different loneliness causes in different cultural contexts. Because mechanisms in the NoDeL framework are suggested to be cross-culturally similar, however, they also offer more specific starting points for interventions (see Table 2).

**Table 2.** Possible Interventions Against Loneliness Based on the Norm Deviations and Loneliness Framework

	Mechanisms							
	Alienation	Inauthenticity	Lower self-worth	Social rejection	Relationship dissatisfaction	Unfulfilled relational needs	Target	Applicability and comments
Broaden range of acceptable behavior/characteristics	x	x	x	x	x	x	Groups	If a social norm puts a large group of people at risk for loneliness; if new social norm is compatible with existing social norms
Promote diversity and tolerance	x	x	x	x	x	x	Groups	
Psychoeducation against pluralistic ignorance	x	x	x	x	x		Groups; individuals	If social norms are systematically misperceived by individuals or groups
Support groups	x	x	x			x	Groups	If multiple individuals deviate from similar characteristics
Individual therapy to change perceptions of deviating from social norms	x	x	x		x		Individuals	If individuals perceive to deviate more than they objectively do
Books, movies, etc. with figures to identify with	x		x		x		Individuals	Particularly if circumstances cannot easily be changed; to quickly relieve loneliness

For one, the most intuitive intervention may be to change social norms that many people deviate from. The goal would be to broaden the range of what is acceptable rather than replace previously normative characteristics or behavior. This is different from many other social-norms interventions, such as *against* littering or excessive alcohol consumption. Returning to the example above (Scoats & Robinson, 2020), an intervention may try to normalize that men display emotional or physical closeness in their friendships without implying that not wanting such closeness in friendships is nonnormative or wrong. New social norms may then be established in media campaigns, intensive group communication, or legal interventions (Bicchieri, 2016). For instance, people with nonnormative yet common characteristics may be portrayed in movies, advertisement, or children’s books (Anderson & Hamilton, 2005; Arias, 2019; Morling & Lamoreaux, 2008; Prot et al., 2015).

Note that such interventions are useful only if many people (perceive to) deviate from the same norm (e.g., to be heterosexual; to be in a partnership). However, in practice, social norms that individuals deviate from are often diverse. Thus, interventions that aim to foster acceptance of diversity and reduce social rejection more generally (Black et al., 2012; Piercy et al., 2002) may be most powerful. For instance, conflict at American schools could be reduced by 30% when influential group members (“social referents”) were encouraged to express opinions against bullying and conflict (Paluck et al., 2016). Rather than replacing specific social norms, such interventions install a metanorm that being different from each other is acceptable. This may have the potential to counteract all mechanisms in the NoDeL framework (see Table 2).

If people feel lonely because they erroneously believe that a characteristic or behavior is normative (i.e., pluralistic ignorance; Allport, 1924), interventions may also need to correct misperceptions. This has, among others, been used to reduce binge drinking among American youths. American university students tend to believe that other students consume more alcohol than they themselves feel comfortable with, which often makes them drink more over time (Prentice & Miller, 1993). Group discussions that allowed to correct such perceptions of social norms could then reduce excessive drinking—particularly among students who were vulnerable to social influence (Schroeder & Prentice, 1998). Similar effects were found in American schools, in which exposure to prevention information about actual social norms regarding alcohol use was related to less high-risk drinking (Perkins et al., 2005). Such interventions may then also help correct erroneous perceptions of being different from others—such as when overestimating others’ sociability on the basis of social media posts.

Note that interventions to change social norms (i.e., the first three interventions in Table 2) should target groups rather than individuals. After all, people continuously update their perceptions of social norms based on (non)verbal behavior they perceive among others (Prentice & Paluck, 2020). This is not to say that every group member needs to be addressed in an intervention: As the study by Paluck et al. (2016) demonstrated, certain group members seem to have a particularly strong influence on group norms. In different research, people lower in the hierarchy of organizations were found to influence perceptions of social norms more strongly than people higher in organizational hierarchies (for an overview, see Prentice & Paluck, 2020). Likewise, acquaintances or remote friends seemed to influence the perception of social norms more strongly than family members or friends. Addressing these group members rather than every individual in a group may then sometimes be sufficient to elicit change.

Interventions addressing mechanisms in the NoDeL framework do not need to be about social norms. For instance, support groups or buddy systems can provide access to relational provisions that are otherwise blocked (Wilkens, 2015). In cultures in which instrumental support is predominantly provided by family, individuals without family may, for example, help each other with moving houses. People who are single in cultures in which touch is widely restricted to romantic partnerships may, among others, find physical closeness during platonic cuddle events. In addition, one may speculate that the belonging that individuals may find in support groups can also ease the alienation, inauthenticity, or lower self-worth that individuals experience in other groups.

The NoDeL framework also suggests possible interventions that can be administered to individuals. Cognitive-behavioral therapy may, for example, help correct erroneous perceptions not to fit in among people who have made experiences of rejection in the past (Feinstein, 2020; Hickin et al., 2021). Such interventions are, however, probably ineffective for individuals whose perceptions to deviate reflect social realities. Individuals who cannot leave social situations in which they do not fit in and/or do not belong to a larger group of people that deviates from the same norm may then find relief in books or movies with characters that they can identify with (Heu, Hansen, et al., 2021).

### Limitations and future directions

The NoDeL framework is built on laypeople’s own perceptions that deviating from social norms had caused their loneliness (Heu, Hansen, et al., 2021) and aligns with previous theorizing (e.g., Akhter-Khan et al., 2023; de Jong Gierveld & Tesch-Römer, 2012; Johnson & Mullins, 1987; Meyer, 2003; Perlman & Peplau, 1981; Qualter et al., 2015) and with converging findings from multiple empirical studies. This empirical evidence, however, is cross-sectional and does, therefore, not allow to test the causal pathways proposed in the NoDeL framework. For instance, as much as norm deviations may cause loneliness, feeling lonelier may make people lose interest in aligning with social norms: Lonely people have been suggested to not only achieve but also enjoy alignment with others’ behavior or emotion less than nonlonely people (Shamay-Tsoory et al., 2019). In addition, previous studies were widely conducted in only two cultural samples, implying that differences in results may not be due to social norms of interest. Longitudinal studies with samples from multiple social contexts and measurement of respective social norms would therefore be better suited to test the NoDeL framework.

Previous research has mostly focused on statistical norms (e.g., median income among people of similar sociodemographic background; e.g., Hawkley et al., 2020) or societally salient and categorical norms (e.g., being unemployed or part of a sexual minority). However, subtle deviations from social norms may also be relevant to study given that lonely people seem particularly sensitive to social rejection (Spithoven et al., 2017). For example, people who had experienced long-lasting loneliness reported having felt lonely when not sharing others’ interests, perceptions, or emotions (Heu, Hansen, et al., 2021). Such vague feelings of not fitting in can be difficult to detect, understand, and, accordingly, cope with (Antonovsky & Sagy, 1986; Williams, 2002). Unlike defined and recognized minorities, people who deviate from subtle norms may, for example, lack a supporting community. To explain the emergence and perpetuation of chronic loneliness, future research should thus also consider subtle norm deviations.

Whereas the NoDeL framework focuses on negative implications of deviating from social norms, previous theorizing suggests that human beings also have a need to feel distinct from others (Brewer, 1991; Hornsey & Jetten, 2004). The NoDeL framework specifies that only norm deviations that are psychologically/socially relevant or crucial for the fulfillment of relational needs should have negative implications for loneliness. Other deviations from group characteristics may hence offer opportunities for more positive distinctiveness. Furthermore, deviating from a characteristic that is normative in a majority group but that includes individuals in a subgroup or different group (optimal distinctiveness theory; Hornsey & Jetten, 2004; Leonardelli et al., 2010) may be experienced more positively than being the only person who is different. Finally, just as certain individual experiences or characteristics can increase the sensitivity to norm deviations, others may protect from experiencing them as negative—and thus allow for positive interpretations of being different. Inclusion and belonging in a (different) group, acceptance by caregivers in childhood, a supportive close friendship or partnership, or genetic disposition, for example, seem to protect from loneliness (e.g., for an overview, see Heu, Hansen, et al., 2021) and may thus reduce negative implications of not fitting in. Future research may then examine which characteristics typically become psychologically or socially relevant and in which specific situations and by whom deviations from social norms are perceived positively rather than as excluding.

### Conclusion

The NoDeL framework suggests that deviations from social norms increase the risk for feeling lonely through alienation, inauthenticity, lower self-worth, and social rejection. Deviations from social norms about social relationships may additionally elevate the risk for loneliness through stronger relationship dissatisfaction or unfulfilled relational needs. Given that these mechanisms are suggested to be similar across cultures while social norms vary between social, geographical, and temporal contexts, social norms may act as cultural moderators in the association between individual-level characteristics and loneliness. Thus, the NoDeL framework may help explain loneliness within cultures, allow to analyze loneliness risks across cultures, and hence provide the basis for culture-sensitive interventions in the future.

## Transparency

*Action Editor:* David A. Sbarra

*Editor:* Interim Editorial Panel

## Notes

1. Although the likelihood to feel lonely should increase with each active mechanism in the NoDeL framework, future research may want to examine if effects add up, interact, or replace each other.

2. For instance, deviations from social norms were not significantly correlated with loneliness in an American study (Hawkley et al., 2020). Deviations from norms were operationalized as deviations from the median or mode on variables such as income, work status, or household size among respondents of the same gender and ethnic/minority status and similar age. The authors themselves explained their findings by suggesting that these reference groups may have been too broad and abstract for individuals to compare themselves with. In addition, characteristics such as income or household size may not have been sufficiently psychologically or socially relevant to increase loneliness.

3. Whereas external processes map onto social rejection in the NoDeL framework, concealment maps onto inauthenticity, and self-stigma maps onto lower self-worth.
